# Ovarian tumor microenvironment contributes to tumor progression and chemoresistance

**DOI:** 10.20517/cdr.2024.111

**Published:** 2024-12-17

**Authors:** Adriana Ponton-Almodovar, Samuel Sanderson, Ramandeep Rattan, Jamie J. Bernard, Sachi Horibata

**Affiliations:** ^1^Precision Health Program, Michigan State University, East Lansing, MI 48824, USA.; ^2^Department of Pharmacology and Toxicology, College of Human Medicine, Michigan State University, East Lansing, MI 48824, USA.; ^3^Department of Women’s Health Services, Henry Ford Health System, Detroit, MI 48202, USA.; ^4^Department of Medicine, College of Human Medicine, Michigan State University, East Lansing, MI 48824, USA.; ^5^Cell and Molecular Biology Program, College of Natural Science, Michigan State University, East Lansing, MI 48824, USA.; ^#^Authors contributed equally.

**Keywords:** Ovarian cancer, chemoresistance, tumor microenvironment

## Abstract

Ovarian cancer is one of the deadliest gynecologic cancers affecting the female reproductive tract. This is largely attributed to frequent recurrence and development of resistance to the platinum-based drugs cisplatin and carboplatin. One of the major contributing factors to increased cancer progression and resistance to chemotherapy is the tumor microenvironment (TME). Extracellular signaling from cells within the microenvironment heavily influences progression and drug resistance in ovarian cancer. This is frequently done through metabolic reprogramming, the process where cancer cells switch between biochemical pathways to increase their chances of survival and proliferation. Here, we focus on how crosstalk between components of the TME and the tumor promotes resistance to platinum-based chemotherapy. We highlight the role of cancer-associated fibroblasts, immune cells, adipocytes, and endothelial cells in ovarian tumor progression, invasion, metastasis, and chemoresistance. We also highlight recent advancements in targeting components of the TME as a novel therapeutic avenue to combat chemoresistance in ovarian cancer.

## INTRODUCTION

Ovarian cancer is predicted to be the 6th leading cause of cancer-related death in women in the United States despite only accounting for ~ 2% of female cancer cases expected in 2024^[1]^. The first-line therapy for ovarian cancer consists of platinum-based chemotherapeutic drugs, such as carboplatin or cisplatin, in combination with paclitaxel^[2]^. Recently, the poly (ADP-ribose) polymerase (PARP) inhibitors olaparib^[3,4]^ and niraparib^[5]^, as well as the vascular endothelial growth factor (VEGF) inhibitor bevacizumab^[4]^, have been used as maintenance therapies for ovarian cancer. However, 80% of ovarian cancer patients develop resistance to treatment through various complex mechanisms, utilizing transporters^[6]^, DNA repair pathways^[7]^, and evading apoptosis^[8,9]^. Interestingly, growing evidence supports that the contribution of tumor microenvironment (TME) plays a major role in chemoresistance. Cancer cells have the capacity to adapt their metabolism in response to stress conditions and environmental demands^[10,11]^. The metabolic adaptation of cancer cells is important for switching between different modes of energy production and communicating signals to the TME. Glucose and glutamine are used for ATP generation and biosynthesis of key metabolites for the rapidly proliferating cells^[12,13]^. Both are directly linked to the tricarboxylic acid (TCA) cycle, replenishing metabolic intermediates in a process called anaplerosis^[12,14]^. The key enzymes of the glutamine metabolism pathway are glutaminase (GLS), glutamate dehydrogenase (GLUD), glutamic oxaloacetic transaminase (GOT), glutamic pyruvic transaminase (GPT), and glutamine synthetase (GS)^[13]^. GLS is responsible for the conversion of glutamine into glutamate, while GLUD, GOT, and GPT transform glutamate into metabolites such as α-ketoglutarate, which is a key intermediate for the TCA cycle^[13]^. GS is responsible for pushing the metabolic flux in the opposite direction by enabling the synthesis of glutamine from glutamate^[13]^. Another key metabolite synthesized from glutamate besides α-ketoglutarate is glutathione, an important antioxidant that protects the cell from oxidative stress by neutralizing reactive oxygen species (ROS)^[13,15]^. Overexpressed GS takes glutamate away from the synthesis of glutathione and redirects it through glutamine into nucleotide biosynthesis^[15]^. Chemoresistance occurrence in ovarian cancer is also due to the increased antioxidant capacity of ovarian cancer cells, such as elevated levels of glutathione^[16]^.

Metabolic adaptations in ovarian cancer affect the response and effectiveness of chemotherapy. Studies have shown that platinum-resistant epithelial ovarian cancer cells have elevated glutamine metabolism, overexpression of GLS, and high levels of glutathione production, which contributes to the resistant phenotype by binding to cisplatin with high affinity to export the drug out of the cell^[17,18]^. Ovarian cancer cells interact with the non-cancer components of their heterogeneous TME, also called the tumor stroma. This includes cancer-associated fibroblasts (CAFs), endothelial cells, adipocytes, and immune cells. These components are associated with the initiation and progression of ovarian cancer, utilizing metabolites and other secreted factors to enhance the tumorigenic potential^[19-21]^. Yang *et al*. comprehensively reviewed these interactions^[20]^, which we are building upon in this review, focusing on the involvement of cell signaling pathways. Previous reviews on this topic have detailed the importance of TME in cancer progression and chemoresistance^[19-22]^. Here, we will describe recent discoveries from the past 5 years (2019-2024) regarding the contributions of the TME, with a focus on CAFs, immune cells, adipocytes, and endothelial cells, to platinum-based drug resistance in ovarian cancer setting [Table 1].

**Table 1:** Factors released by components of the tumor microenvironment affect tumor progression and chemoresistance

**Components**	**Factors**	**Process**
CAFs	CXCL14^[23]^ miR-296-3p^[24]^ CCL5^[25]^ miR-98-5p^[26]^ IFN1β^[27]^ Wnt5a^[28]^ CXCL12^[29]^ Jagged 1^[30]^	Promotes chemoresistance
	MMP^[22]^	Promotes cancer invasion
	FAP^[20]^ CXCL14^[23]^	Promotes tumor metastasis
TAMs	CXCL16^[31]^ GATA3^[32]^ miR-221-3p^[33]^ CXCL12^[20]^	Promotes chemoresistance
	IL-10^[20]^ CCL17^[20]^ CCL22^[20]^	Promotes tumor growth
Cancer-associated adipocytes	IGF-1^[34]^ Adipokines^[35]^ Free Fatty Acids^[36]^	Promotes chemoresistance
	MCP-1^[37]^ TIMP1^[37]^	Promotes tumor metastasis
Cancer-associated endothelial cells	VEGF^[38]^	Induces angiogenesis
	VEGF^[39]^ Jagged 1^[40]^	Promotes chemoresistance

CAFs: Cancer-associated fibroblasts; TAMs: tumor-associated macrophages; CXCLs: C-X-C chemokine ligands; CCLs: C-C chemokine ligands; IFNβ: interferon beta; MMP: matrix metalloproteinases; FAP: fibroblast activation protein; GATA3: GATA binding protein 3; IL-10: interleukin-10; IGF-1: insulin-like growth factor-1; MCP-1: monocyte chemoattractant protein-1; TIMP1: tissue inhibitors of metalloproteinases 1; VEGF: vascular endothelial growth factor.

## CAFs

### Characteristics of CAFs

**Figure 1:**
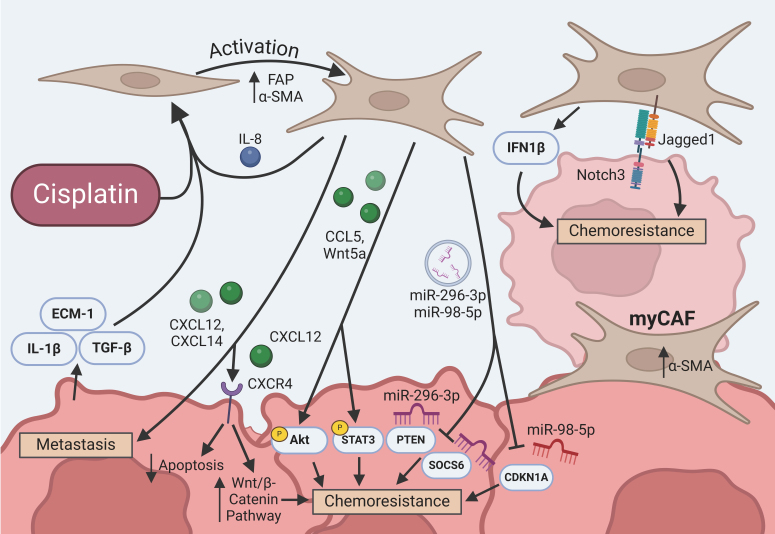
Intercellular crosstalk between CAFs and ovarian cancer cells. Cancer cells, CAFs, and cisplatin can all contribute to the activation of normal fibroblasts. CAFs secrete factors and exosomes to alter key pathways involved in chemoresistance. Lastly, CAFs communicate through cell-cell contact, and certain subtypes such as myCAFs are known for their close proximity. CAFs: Cancer-associated fibroblasts; myCAFs: myofibroblastic cancer-associated fibroblasts; FAP: fibroblast activation protein α; α-SMA: α-smooth muscle actin; ECM-1: extracellular matrix protein-1; TGF-β: transforming growth factor-β; IFN1: interferon 1; IL-1β: interleukin-1β; STAT3: signal transducer and activator of transcription 3; PTEN: phosphatase and tensin homolog; SOCS6: suppressor of cytokine signaling; CDKN1A: cyclin-dependent kinase inhibitor 1A; Akt: protein kinase B.

CAFs are centrally agreed upon as fibroblasts that are activated by signals from cancer cells and promote tumor survival^[41,42]^. Thus, CAFs share many markers with normal activated fibroblasts, including fibroblast activation protein α (FAP), α-smooth muscle actin (α-SMA), and vimentin^[41]^ [Figure 1]. CAFs are the principal constituent of the TME, maintaining the extracellular matrix (ECM) and tumor stroma to support the growth of cancer cells^[41]^. CAFs secrete matrix proteins such as collagen, laminin, and fibronectin alongside factors such as matrix metalloproteinases (MMP), which are involved in tissue remodeling and angiogenesis, and tissue inhibitors of metalloproteinases (TIMPs), which are endogenous MMP regulators^[43]^. To create CAFs, resting fibroblasts are activated through signaling from cancer cells or other CAFs [Figure 1]^[44,45]^. CAF formation is also induced by a hypoxic environment through the increased presence of microRNA-210 and transforming growth factor-β (TGF-β)^[22]^. Extracellular matrix protein-1 (ECM1) secreted by ovarian cancer cells promotes CAF activation by increasing the expression of FAP and α-SMA [Figure 1]^[46]^. Interestingly, platinum-drug treatment also increases FAP expression, induces hypoxia, and alters the metabolism of normal fibroblasts to promote transformation^[47]^.

### Effect of CAFs on cancer invasion and metastasis

The proteolytic activity of the MMPs secreted by CAFs promotes cancer invasion by degrading the ECM in the microenvironment^[22]^. CAFs utilize their increased expression of FAP, which acts as a protease, to further degrade the matrix and promote tumor metastasis^[20]^. Overexpression of CAF-induced chemokine ligands (CXCL12 and CXCL14) has been associated with cancer growth and metastasis [Figure 1]^[20,23]^. CXCL14 promotes glycolysis, tumor growth, and metastasis by interacting with the enzyme 6-phosphofructo-2-kinase/Fructose-2,6-biphosphatase 2 (PFKFB2)^[23]^. Ovarian cancer cells also secrete high levels of interleukin-1β (IL-1β) and TGF-β, which induce the activation of CAFs^[48,49]^ and thereby promote invasion and metastasis [Figure 1]. A recent study showed that TGF-β also promotes the expression of cation amino acid transporter solute carrier family 7 member 1 (SLC7A1) in CAFs^[50]^. CAFs with high SLC7A1 promote invasion, migration, and metastasis in ovarian cancer cells^[50]^. SLC7A1 is also expressed in tumor cells and promotes epithelial-to-mesenchymal transition (EMT), the process where epithelial-like cancer cells exhibit mesenchymal phenotype^[50]^. This is indicated through decreased expression of the epithelial marker E-cadherin and increased expression of the mesenchymal marker N-cadherin^[50]^. Overall, CAFs and ovarian cancer cells work in tandem. As ovarian cancer cells promote CAF activation, those activated CAFs secrete factors that promote further activation alongside tumor invasion and metastasis.

### CAF-mediated chemoresistance

Recently, many advancements have been made to improve our understanding of how CAFs promote platinum resistance in ovarian cancer^[41,42]^. CAFs in the TME induce a shift to a more fibrotic environment, changing the mechanical properties of the ECM and altering the efficacy of anti-cancer drugs^[22]^. Furthermore, the occurrence of angiogenesis causes hypervascularization and an increase in the activation of CXCR4 [Figure 1]^[22]^. This promotes phosphoinositide 3-kinases (PI3K), Rho factor, and mitogen-activated protein kinase signaling, which reduce direct drug interaction with the target cancer cells^[22]^. Eckert *et al*. reviewed that platinum-based drugs also alter the CAF secretion factors or secretome^[47]^. Here, we will elaborate on how CAF-secreted extracellular vesicles, myofibroblastic CAFs (myCAFs), and utilization of Wnt and Notch signaling by CAFs contribute to chemoresistance.

#### Extracellular vesicles

CAFs utilize exosomes to induce chemoresistance in ovarian cancer^[24,25]^. Exosomes are a type of extracellular vesicle, small membrane-bound bubbles that act as an intercellular communication system by merging with the cell membrane^[51]^. Platinum-based chemotherapy can induce CAFs to secrete exosomes containing microRNAs, short RNA sequences that bind to the 3’UTR of mRNAs to prevent translation. CAFs have been shown to deliver miR-296-3p, which targets the mRNAs of phosphatase and tensin homolog (PTEN) and suppressor of cytokine signaling 6 (SOCS6)^[24]^ [Figure 1]. PTEN is a negative regulator of the PI3K/protein kinase B (Akt) survival pathway, and its inhibition subsequently increases cell proliferation, metastasis, and cisplatin resistance^[24,52]^. SOCS6 also functions as a negative regulator, acting in the signal transducer and activator of transcription 3 (STAT3) pathway^[24]^. By inhibiting SOCS6, the STAT3 pathway is activated further, increasing cancer progression and cisplatin resistance^[53]^. miR-98-5p is also transported by CAF exosomes and targets the mRNA for cyclin-dependent kinase inhibitor 1A (CDKN1A), a cell cycle regulator that, when inhibited, causes resistance [Figure 1]^[26]^. Overall, exosomes are an effective way for CAFs to communicate within the TME and induce resistance to platinum-based chemotherapies in ovarian cancer.

Another study found that the chemokine (C-C motif) ligand 5 (CCL5) secreted from CAFs increases STAT3 and Akt phosphorylation^[25]^. Both STAT3 and Akt are activated when phosphorylated, and their respective pathways both promote cisplatin resistance^[25]^. Additionally, CAFs secrete the exocrine protein periostin, which increases Akt phosphorylation^[54]^. Interestingly, cisplatin-induced DNA damage was found to instigate the transfer of DNA fragments from ovarian cancer cells to CAFs^[27]^. The CAFs then detect the damage through the binding of DNA to cyclic GMP-AMP synthase (cGAS), which activates the stimulator of interferon genes (STING) inflammation pathway^[27,55]^. This pathway causes the CAFs to release interferon beta 1 (IFN1B), an inflammatory cytokine that increases cisplatin resistance by upregulating DNA repair mechanisms, preventing apoptosis, and increasing proliferation^[27]^. These unique communication methods facilitated by CAFs all work to promote ovarian cancer survival in the presence of platinum-based chemotherapy.

#### myCAFs

CAFs can be further divided into distinct subtypes, one of which is called myCAFs. MyCAFs are characterized by close proximity to tumor cells and high expression of α-SMA [Figure 1]. This subtype has been shown to promote chemoresistance in ovarian clear cell carcinoma (OCC)^[56]^. OCC cells release platelet-derived growth factor (PDGF), which binds to its receptor on myCAFs^[56]^. The myCAFs then induce the downstream release of hypoxia-inducible factors 1-α (HIF1-α) in the OCC cells^[56]^. HIF-1-α is traditionally activated during hypoxia, where its continual degradation is halted to allow it to function^[57]^. Activation of HIF-1-α promotes chemoresistance through several mechanisms in ovarian cancer, including inhibition of p53 to prevent tumor suppression and increased autophagy to conserve energy and prevent apoptosis^[57,58]^. Thus, myCAFs contribute to chemoresistance via HIF-1-α.

#### Wnt signaling

The Wnt signaling pathway is involved in the progression, therapy resistance, and invasion of many cancers, including ovarian cancer^[59-61]^. Its canonical form is the most well-characterized, and involves a Wnt ligand binding to the Frizzled receptor and receptor tyrosine kinase-like orphan receptor 1 or 2 (ROR1/2) coreceptors^[59]^. This stops the continuous degradation of the β-catenin protein^[59]^. This allows β-catenin to enter the nucleus and recruit transcription factors to promote processes such as EMT and cancer stem cell dedifferentiation^[59]^. The non-canonical Wnt pathways are defined by their lack of β-catenin involvement, but are still initiated by Wnt binding to Frizzled and ROR1/2^[59]^.

Non-canonical Wnt signaling is utilized in CAF-to-ovarian cancer communication and induction of chemoresistance. CAFs near ovarian cancer cells increase chemoresistance and dedifferentiation to cancer stem cells (CSC) by releasing the Wnt5a ligand^[28]^. CSCs are cancer cells that act as stem cells for the tumor by differentiating and rapidly dividing to increase or maintain their size^[62]^. CSCs have been shown to resist chemotherapy in many different cancers, including ovarian cancer^[62]^. The CAF-mediated transformation of ovarian cancer cells to CSCs involves non-canonical Wnt5a binding to ROR1 and ROR2^[28,63]^. Wnt5a binding to ROR2 activates the protein kinase C (PKC) cAMP response element binding protein 1 (CREB1) pathway (PKC/CREB1), which induces dedifferentiation into CSCs and chemoresistance^[28]^. Binding to ROR1 activates the Akt/extracellular signal-regulated kinase (ERK)/STAT3 pathway, which promotes chemoresistance, CSC development, and EMT^[27]^.

Additionally, CAFs release stromal-derived factor-1α (CXCL12), which binds to the CXCR4 receptor on ovarian cancer cells and increases resistance to cisplatin-induced apoptosis^[29]^. This causes activation of the canonical Wnt/β-catenin pathway, which increases EMT and cisplatin resistance [Figure 1]^[29,64]^. In malignant peripheral nerve sheath tumors, CXCR4 activates this pathway by repressing glycogen synthase kinase-3 β (GSK-3β), one of the kinases that phosphorylates β-catenin to mark it for degradation, but whether that is the same in ovarian cancer remains to be elucidated^[65]^.

#### Notch3 pathway

Notch3 appears to be particularly important in ovarian cancer, as it has the greatest increase in Notch3 expression compared to every cancer in the TCGA database^[66]^. The Notch3 pathway is heavily involved in CSC development, proliferation, and chemoresistance of different drugs for multiple cancers and is often activated through cell-cell contact^[66]^. CAFs express the Jagged 1 ligand, which binds and activates the Notch3 pathway, resulting in an increase in growth, EMT, and chemoresistance [Figure 1]^[30]^. This also induces the release of vascular endothelial growth factor A (VEGFA), a known promoter of angiogenesis that also causes further dedifferentiation of CSC^[30]^. CAFs release the chemokine interleukin-8 (IL8), also called CXCL8, which binds to CXCR1/2 receptors and promotes the transformation of normal fibroblasts^[67]^. IL-8 binding to CXCR1/2 also promotes CSC development and cisplatin resistance in ovarian cancer by activating Notch3^[67]^. However, the mechanism of Notch3 activation through this chemokine is still unclear^[67]^.

## CANCER-ASSOCIATED IMMUNE CELLS

**Figure 2:**
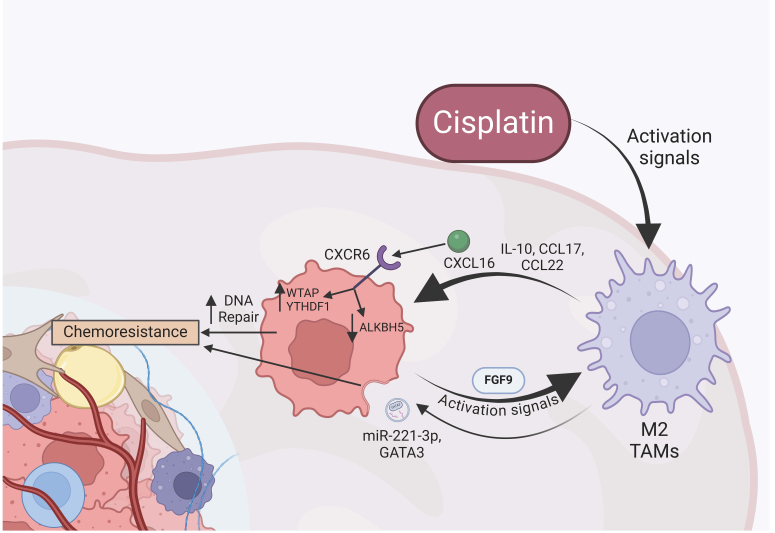
Intercellular crosstalk between the predominant M2 TAMs and ovarian cancer cells. Cisplatin activates M2 TAMs to secrete exosomes and cytokines (IL-10, CCL17, CCL22) that activate chemoresistance mechanisms in ovarian cancer cells. Specifically, the cytokine CXCL16 interacts with the CXCR6 receptor, increasing WTAP and YTHDF1 and decreasing ALKBH5 expression. This results in increased DNA repair mechanisms and leads to chemoresistance. WTAP: Wilms tumor 1-associated protein; YTHDF1: YTH N6-methyladenosine RNA binding protein F1; ALKBH5: alkB homolog 5; CXCR6: C-X-C chemokine receptor type 6; CXCL16: C-X-C chemokine ligand 16; IL-10: interleukin-10; CCLs: chemokine (C-C motif) ligands; FGF9: fibroblast growth factor-9; TAMs: tumor-associated macrophages.

Immune cells are another critical component of the TME. Ovarian cancer cells directly interact with immune cells to form an immunosuppressive TME, evading detection and destruction^[68]^. The main immune cells that play a role in ovarian cancer are macrophages and tumor-associated macrophages (TAMs)^[69]^. Classically activated macrophages (M1) associated with cancer cells are pro-inflammatory with tumor suppression and cytotoxicity activity. M1 macrophages secrete cytokines, such as interleukin 1 (IL-1), IL-12, tumor necrosis factor α (TNF-α), and CXCL12^[20]^. Cisplatin promotes a tumor-suppressive immune response by recruiting M1 macrophages and tumor-specific CD8+ T cells^[70]^. Conversely, the alternatively activated macrophages (M2) are the predominant macrophages in ovarian cancer and promote tumor growth through the secretion of immunosuppressive cytokines [e.g., IL-10, chemokine (C-C motif) ligand 17 (CCL17), CCL22] [Figure 2]^[20]^. In addition, fibroblast growth factor-9 (FGF-9) is secreted from ovarian cancer cells to induce M2 polarization of TAMs [Figure 2]^[71]^. M2 macrophages are also associated with remodeling the ECM by producing MMPs. In the TME, the high levels of cytokines, such as IL-4 and IL-13, promote the differentiation of monocytes to M2 macrophages^[20]^. This maintains the immunosuppressive behavior that allows tumor survival and progression by producing signaling molecules that participate in tumorigenesis, metastasis, and angiogenesis^[20]^.

### TAMs

TAMs can have either an M1 or M2 phenotype but are predominately M2 and act to promote cancer metastasis and chemoresistance^[72]^. TAMs secrete the CXCL16 cytokine, which binds to the CXCR6 receptor on ovarian cancer cells [Figure 2]^[31]^. This binding increases the expression of Wilms tumor 1-associated protein (WTAP) and YTH N6-Methyladenosine RNA Binding Protein F1 (YTHDF1), both of which are involved in N6-methyladenosine (m6A) RNA methylation^[31]^. M6A RNA methylation is a type of post-transcriptional RNA modification that is mediated by protein complexes called “writers”, “readers”, and “erasers”^[31,73,74]^. The writers, which includes WTAP, methylate the RNA while the readers, such as YTHDF1, interpret the modifications and direct the RNA^[31,73,74]^. The erasers, α-ketoglutarate-dependent dioxygenase (FTO) and alkB homolog 5 (ALKBH5), remove the modifications^[73,74]^. The expression of these components is altered in cancer to dysregulate downstream pathways and promote tumor survival^[74]^. In ovarian cancer, activating the CXCR6 receptor increases the expression of the WTAP writer and YTHDF1 reader while decreasing the ALKBH5 eraser, suggesting increased m6A methylation^[31]^. This then induces chemoresistance through an increase in DNA repair, which counteracts the toxicity of platinum-based chemotherapy, and a decrease in expression of pro-apoptotic proteins^[31]^. However, the mechanism connecting CXCR6 activation and increased m6A methylation remains to be determined^[31]^.

#### TAMs use of extracellular vesicles

Like CAFs, TAMs use exosomes for intercellular communication with ovarian cancer cells, promoting tumor progression and chemoresistance. GATA binding protein 3 (GATA3) is a transcription factor that is transported into ovarian cancer cells through EVs and increases the expression of CD24 [Figure 2]^[32]^. CD24 then promotes the expression of the Siglec-10 receptor and increases chemotherapy resistance by downregulating the apoptosis regulators B-cell lymphoma (BCL-2) and caspase-3^[32]^. TAMs in ascites also use exosomes to transport microRNAs. TAMs export miR-221-3p, which targets the expression of A disintegrin and metalloproteinase with thrombospondin motifs 6 (ADAMTS6) in ovarian cancer [Figure 2]^[33]^. This reduction in ADAMTS6 causes an upregulation of the epidermal growth factor receptor (EGFR)/TGF-β/Akt pathway, which promotes EMT and CSC-related genes alongside the multidrug resistance (MDR) gene, contributing to chemoresistance^[33]^. TAMs and CAFs using exosomes to induce chemoresistance present a potential for therapies targeting this communication mechanism to inhibit the action of both cell types.

## CANCER-ASSOCIATED ADIPOCYTES

**Figure 3:**
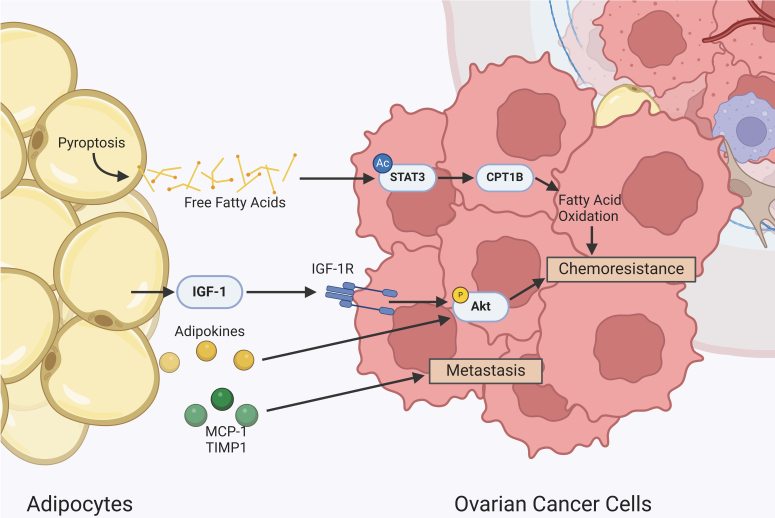
Intercellular crosstalk between cancer-associated adipocytes and ovarian cancer cells. Adipocyte secretions promote metastasis and chemoresistance. Adipocytes release free fatty acids, which are taken up by the tumor cells and are metabolized through fatty acid oxidation, which promotes chemoresistance. STAT3: Signal transducer and activator of transcription 3; CPT1B: carnitine palmitoyltransferase 1B; IGF-1: insulin-like growth factor-1; TIMP1: tissue inhibitors of metalloproteinases 1; MCP-1: monocyte chemoattractant protein-1; Akt: protein kinase B.

Several studies have found a close relationship between adipocytes and ovarian cancer^[75,76]^. Adipocytes produce fatty acids, cytokines, and chemokines such as IL-6, IL-8, monocyte chemoattractant protein-1 (MCP-1), and TIMP1, which promote cancer growth and metastasis [Figure 3]^[37]^. The proximity between the ovaries and the omentum generates a predisposition for this fat region to become the primary site of ovarian cancer metastasis^[75]^. It has been reported that ovarian cancer cells modify their lipid metabolism by upregulating fatty acid-binding protein 4 (FABP4) in the adipocyte-cancer cell interface at omental metastases^[75]^. This promotes fatty acid uptake from the neighboring adipocytes to stimulate tumor progression^[75]^. Inhibiting FABP4 decreases metastasis and increases sensitivity to carboplatin, indicating it plays a role in chemoresistance and is a potential target for new therapies^[77]^. Additional members of the FABP protein family, FABP5 and FABP (PM), also contribute to chemoresistance by increasing fatty acid uptake^[78]^.

Insulin-like growth factor-1 (IGF-1) secreted from adipocytes promotes chemoresistance by binding to IGFR on OCC and can be targeted to increase cisplatin sensitivity [Figure 3]^[34]^. This further indicates that adipocytes are critical components of the ovarian cancer TME, strongly influencing its metastatic and chemoresistant properties. A clinical and genomic data analysis showed that higher expression levels of genes related to obesity or lipid metabolism, particularly fatty acid receptor CD36 and TGF-β, are associated with poor prognosis^[79]^. Specifically, CD36 participates in angiogenesis regulation and fatty acid uptake by ovarian cancer cells, promoting cell migration and proliferation^[79]^. Liu *et al.* investigated the contribution of obesity to ovarian cancer metastasis and found an increased expression of sterol regulatory element-binding protein 1 (SREBP-1)^[69]^. SREBP-1 is a transcription factor involved in fatty acid synthesis and lipid homeostasis^[69,80]^. Thus, high expression levels are associated with increased lipogenesis gene transcription^[69,80]^. Its association with enhanced ovarian cancer tumor burden has led to the investigation of its properties as a therapeutic target for ovarian cancer in obese women^[81]^.

### Adipocytes and inflammation

Moreover, obesity is characterized by producing chronic inflammation. Hence, there is an accumulation of immune cells secreting cytokines alongside adipokines secreted by adipocytes^[81]^. This creates a highly immunosuppressive microenvironment that provides a proliferative advantage for ovarian tumor growth. A recent study found that ovarian cancer cells also release IL-6 and IL-8 to induce pyroptosis, inflammation-based apoptosis, in adipocytes^[36]^. The death of these adipocytes causes the release of free fatty acids, which are taken up by ovarian cancer cells and cause an upregulation of acetylated STAT3 and carnitine palmitoyltransferase 1B (CPT1B) [Figure 3]^[36]^. Acetylated STAT3 increases the expression of CPT1B^[82]^, which is an important enzyme in fatty acid oxidation^[83]^. An increase in fatty acid oxidation induces chemoresistance [Figure 3]^[36,78,84]^.

Cancer-associated adipocytes are critical for chemoresistance in ovarian cancer^[35]^. Their secreted adipokines activate the pro-survival Akt signaling pathway that facilitates cancer persistence^[35]^. Cisplatin has been associated with increased lipolysis in adipocytes while inhibiting lipogenesis^[85]^. This leads to elevated fatty acid secretion from adipocytes, serving as an energy source for ovarian cancer cells. Amino acid metabolism can also be associated with acquired chemoresistance in ovarian cancer. Chemoresistant epithelial ovarian cancer has increased dependence on glutamine as an energy source, which fuels the TCA cycle. It is currently being investigated as a glutamine-mediated form of platinum resistance^[86]^.

Ahmed *et al.* evaluated the metabolic plasticity of chemotherapy-treated ovarian cancer cells and demonstrated that chemotherapy stimulates oxidative phosphorylation-mediated lipid metabolism^[19]^. Several enzymes were upregulated, including the pyruvate dehydrogenase phosphatase regulatory subunit (PDPR), which inhibits acetyl-CoA production from pyruvate^[19]^. This suggests that chemotherapy contributes to metabolic reprogramming that fuels the TCA cycle, allowing for direct oxidative phosphorylation. The correlation and implications between cancer-associated adipocytes and amino acid metabolism in chemoresistance need further investigation.

## CANCER-ASSOCIATED ENDOTHELIAL CELLS

**Figure 4:**
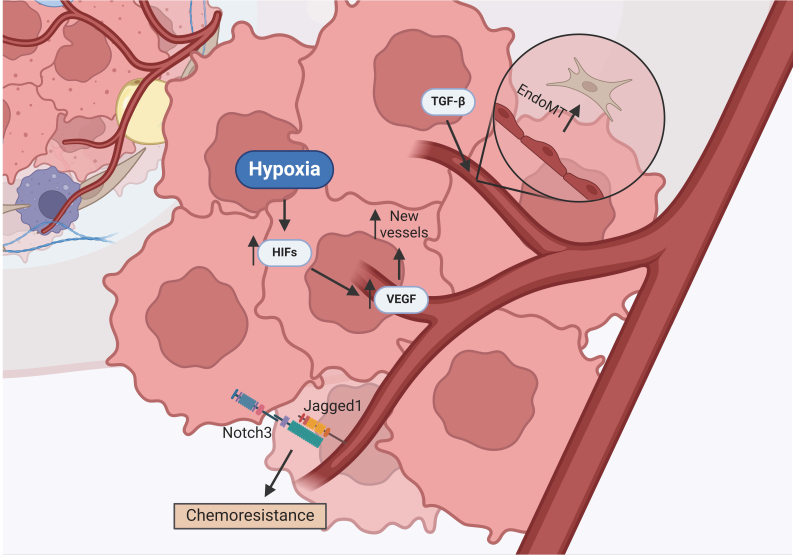
Crosstalk between cancer-associated endothelial cells and ovarian cancer cells. TGF-β from tumor cells induces EndoMT to transform endothelial cells into CAFs. Hypoxic conditions within the tumor cause the release of hypoxia-induced factors, which promote angiogenesis. Activation of the Akt pathway in endothelial cells causes increased expression of the Jagged1 ligand, which triggers activation of the Notch3 pathway through cell contact, promoting chemoresistance. TGF-β: Transforming growth factor-β; CAFs: cancer-associated fibroblasts; HIFs: hypoxia-inducible factors; VEGF: vascular endothelial growth factor; EndoMT: endothelial-to-mesenchymal transition.

Endothelial cells from the vascular endothelium become a key player in the TME. These cells aid in maintaining metabolic homeostasis, transporting metabolites and oxygen, and participating in angiogenesis, i.e., the formation of new blood vessels^[20,87,38]^. As the tumor grows, it becomes hypoxic and acidic, leading to the activation of hypoxia-inducible factors (HIFs) [Figure 4]^[38]^. HIFs regulate the initiation of vessel sprouting, where the endothelial cells secrete proangiogenic factors, such as VEGF [Figure 4]^[38]^. VEGF is an angiogenesis activator and induces the migration of endothelial cells toward the tumor to form new vessels [Figure 4]^[38]^. In ovarian cancer, studies have shown that expression of VEGF is elevated at later stages and is associated with cisplatin resistance^[39]^.

Newly formed vessels usually lack cell-to-cell connections, facilitating intravasation, a process where the cancer cells enter the vasculature^[38]^. Thus, cancer-associated endothelial cells are crucial in cancer migration and metastasis. In addition, these cells show high plasticity, which facilitates endothelial-to-mesenchymal transition (EndoMT), wherein they become CAFs [Figure 4]^[38]^. This process is mediated by TGF-β, which is also associated with cancer cell invasion^[38]^.

Similar to CAFs, endothelial cells are able to cause resistance to chemotherapy through crosstalk with ovarian cancer cells. Activation of the PI3K/Akt pathway in endothelial cells promotes angiogenesis and increases expression of the Jagged1 ligand [Figure 4]^[40]^. Like in CAFs, the Jagged1 ligand then binds to and activates the Notch3 pathway in OCC, which increases resistance to cisplatin [Figure 4]^[40]^.

Altogether, the dynamic TME affects the metabolic behavior of ovarian cancer by promoting its survival, progression, and resistance to chemotherapy. In contrast, ovarian cancer cells can subvert the metabolic activity of these neighboring cells for their proliferative advantage. It has been suggested that this metabolic reprogramming directly influences the effectiveness of chemotherapy.

## TARGETING THE TME

Several therapeutic strategies that target the TME components include anti-angiogenesis therapy inhibiting VEGF and its receptor VEGFR, as well as immune checkpoint inhibitors such as anti-CTLA-4 and anti-PD1/PD-L1^[20,88,89]^. Over the last 5 years, many advancements have been made in targeting CAFs and TAMs to reduce chemoresistance in ovarian cancer. Therefore, our focus will be primarily on them.

Due to the high population of CAFs in the ovarian TME, CAF-targeted therapy is being used^[20]^. The STING inhibitor H-151 increases sensitivity by blocking downstream IFNB1 production in CAFs, which, as previously mentioned, induces platinum resistance^[27]^. Ripretinib is an U.S. Food and Drug Administration (FDA)-approved drug for treating advanced-stage gastrointestinal stromal tumors, acting as an inhibitor of tyrosine kinases to block downstream pathways that cancer cells rely on for survival^[90]^. Ripretinib has also shown high efficacy in killing CAFs and lowering resistance in ovarian cancer by blocking the PDGF receptor^[56]^. Ripretinib acts synergistically with carboplatin and presents itself as a promising combination therapy^[56]^.

The same study that showed ECM1 secretion by ovarian cancer promotes CAF development and chemoresistance also tested the effects of the algae-extracted compound Wentilactone A^[56]^. Wentilactone A prevents the phosphorylation of inhibitor of κB kinase (IKK) and inhibitor of nuclear factor kappa B (IκB), which act upstream of NF-κB^[56]^. This inhibits the activation of NF-κB, causing a downstream reduction in ECM1^[56]^. Overall, Wentilactone A reduces ECM1 secretion to reverse cisplatin sensitivity in ovarian cancer^[56]^.

Similarly, other strategies are developed to target TAMs in the TME. Triptolide (TPL) is a biologically active diterpene triepoxide that has anti-inflammatory effects^[91]^. TPL has been shown to reduce the proliferation, survival, migration, and invasion of cisplatin-resistant ovarian cancer, and when given alongside cisplatin, prevents TAMs from shifting to the M2 phenotype^[92]^. A recent study showed enhanced stabilization of the FGF-9 mRNA, which increases secretion, through the interaction between the non-coding, circular RNA circITGB6 and the m6A methylation reader insulin-like growth factor 2 mRNA-binding protein (IGF2BP)^[71]^. Additional treatments act by suppressing macrophage recruitment through targeting kinase receptors such as colony-stimulating factor 1 receptor (CSF-1R), which is expressed on ovarian cancer cells^[20]^. This reduces the infiltration of M2 macrophages and increases cisplatin sensitivity^[20]^.

## CONCLUSION

Many advancements have been made to improve our understanding of the TME and how it affects chemoresistance in ovarian cancer. Specifically, many studies have been published focusing on how CAFs, TAMs, and adipocytes induce platinum resistance, making them exciting avenues for therapeutic intervention. Approximately 80% of ovarian cancer patients develop tumor recurrence and chemoresistance^[9]^. It is critical that new resistance mechanisms continue to be discovered and new therapies are developed to target these systems both within the cancer cells and in their extracellular TME.

## DECLARATIONS

### Copyright

© The Author(s) 2024.
